# Dual-transgenic BiFC vector systems for protein-protein interaction analysis in plants

**DOI:** 10.3389/fgene.2024.1355568

**Published:** 2024-03-08

**Authors:** Piaojuan Chen, Meiling Ye, Yadi Chen, Qin Wang, Qiongli Wang, Ming Zhong

**Affiliations:** College of Agriculture, Basic Forestry and Proteomics Research Center, Fujian Agriculture and Forestry University, Fuzhou, China

**Keywords:** protein-protein interaction (PPI), bimolecular fluorescence complementation (BiFC), EYFP, mVenus, mRFP1Q66T, mCherry

## Abstract

Protein-protein interaction (PPI) play a pivotal role in cellular signal transduction. The bimolecular fluorescence complementation (BiFC) assay offers a rapid and intuitive means to ascertain the localization and interactions of target proteins within living cells. BiFC is based on fluorescence complementation by reconstitution of a functional fluorescent protein by co-expression of N- and C-terminal fragments of this protein. When fusion proteins interact, the N- and C-terminal fragments come into close proximity, leading to the reconstitution of the fluorescent protein. In the conventional approach, the N-terminal and C-terminal fragments of the fluorescent protein are typically expressed using two separate vectors, which largely relies on the efficiency of the transformation of the two vectors in the same cells. Furthermore, issues of vector incompatibility can often result in loss of one plasmid. To address these challenges, we have developed novel dual-transgenic BiFC vectors, designed as *pDTQs*, derived from the previously published *pDT1* vector. This set of BiFC vectors offers the following advantages: 1) Both fluorescent fusion proteins are expressed sequentially within a single vector, enhancing expression efficiency; 2) Independent promoters and terminators regulate the expression of the two proteins potentially mitigating vector compatibility issues; 3) A long linker is inserted between the fluorescent protein fragment and the gene of interest, facilitating the recombination of the fused fluorescent protein into an active form; 4) Four distinct types of fluorescent proteins, namely, EYFP, mVenus, mRFP1Q66T and mCherry are available for BiFC analysis. We assessed the efficiency of the *pDTQs* system by investigating the oligomerization of *Arabidopsis* CRY2 and CRY2-BIC2 interactions in *N. benthamiana*. Notably, the *pDTQs* were found to be applicable in rice, underscoring their potential utility across various plant species.

## 1 Introduction

Climate change poses a significant and escalating threat to tropical plants both in the present and the foreseeable future. Rice (*Oryza sativa L.*), as a tropical economic crop, is the most widely consumed staple food for a large part of the world’s human population, especially in Asia (Samal et al., 2018). However, climate change, which influences the regularity and level of hydrological fluctuations, is a major threat to agriculture, particularly in developing nations, and causes various abiotic stresses for tropical plants (Turral et al., 2011). The development and application of climate-resilient varieties are urgent matters for scientists. To address this issue, scientists have employed multi-omics approaches, including genomics, transcriptomics, phenomics, metabolomics, and proteomics. These approaches are crucial for understanding biological processes, heavily relying on identifying interacting protein partners and accurately visualizing protein-protein interactions (PPI). Protein interaction networks play a crucial role in processing environmental cues, regulating metabolism, and guiding development in all organisms. In addition, PPI serves as the fundamental basis for complex cellular signaling. More and more techniques have been developed to study PPI, including mass spectrometry, which is widely used in omics research. Mass spectrometry offers convenience for studying plant development, metabolism, and environmental responses (Aebersold and Mann, 2003). However, some potential interacting proteins identified by mass spectrometry need to be validated using different techniques, such as the yeast two-hybrid system (Ehlert et al., 2006), co-immunoprecipitation assay (Comai, 2003; Lee, 2007), protein fragment complementation assays (Chen et al., 2008), and fluorescence resonance energy transfer (FRET) (Miyawaki et al., 1997; Lalonde et al., 2008). Although all of the above techniques are used to identify interacting proteins and reveal the composition of complexes and possible molecular resolution structure, it is challenging to monitor the dynamics of interaction and localization *in vivo* in real time, which is necessary to understand how proteins function at the cellular, tissue, and organism levels.

Bimolecular Fluorescence Complementation (BiFC) assays are powerful tools in molecular biology and cell biology that allow researchers to investigate protein-protein interactions in living cells (Bhat et al., 2006; Kim et al., 2007; Hynes et al., 2008; Kerppola, 2008; Gehl et al., 2009). This technique is based on the principle of splitting a fluorescent protein into two non-fluorescent fragments and fusing each fragment to a protein of interest (Lee et al., 2010; Ohad and Yalovsky, 2010). When these proteins interact with each other, the two fragments come together, reconstituting the fluorescent protein and producing a visible fluorescence signal. This signal serves as an indicator of the interaction between the two proteins, providing valuable insights into various cellular processes (Hu et al., 2002; Grinberg et al., 2004). BiFC assays are primarily used to study the interactions between proteins within living cells. Moreover, BiFC can be employed to determine the subcellular localization of protein complexes (Citovsky et al., 2006; Susperreguy et al., 2011). By tagging the proteins of interest with BiFC fragments and visualizing the resulting fluorescence, researchers can gain insights into where these interactions occur within the cell (Bhat et al., 2006). BiFC also enables the monitoring of dynamic alterations in protein interactions over time. By observing the fluorescence signal in real-time, researchers can assess the temporal aspects of protein interactions in response to various stimuli or conditions (Kerppola, 2006; Concepcion et al., 2009; Shao et al., 2021). Furthermore, BiFC assays can be adapted for high-throughput screening to identify potential drug targets or assess the effects of small molecules on protein interactions (Gehl et al., 2009; Mukherjee et al., 2022). This is particularly valuable in drug discovery and development (Dai et al., 2013; Bellón-Echeverría et al., 2018). However, one significant limitation of BiFC assays is that they are irreversible. Once the fluorescent protein fragments reconstitute and produce a signal, the interaction cannot be reversed or undone (Miller et al., 2015). This means that the assay provides information about the presence of an interaction but does not indicate whether it is transient or stable. BiFC assays can generate false-positive results if the tagged proteins aggregate or if the fluorescent protein fragments interact independently of the proteins of interest (Liu et al., 2014). Careful controls and validation are necessary to distinguish genuine interactions from artifacts. Furthermore, the fusion of protein fragments to the target proteins may interfere with their natural conformation or function. This could lead to alterations in the protein’s behavior, potentially affecting the interaction being studied (Mukherjee et al., 2022). In addition, BiFC assays provide qualitative information about protein interactions (Bais et al., 2023), for example, whether an interaction occurs or not, but do not provide quantitative data about the strength or affinity of the interaction. The efficiency of co-transformation and expression of two BiFC proteins, and the sensitivity of fluorescent protein in BiFC experiments are still worthy of improvement (Frutiger et al., 2019). When studying photoreceptors, it is crucial to choose the appropriate fluorescence type to prevent using the same excitation wavelength for both fluorescent proteins and photoreceptors, as this could inadvertently excite the photoreceptors (Wang et al., 2016; Han et al., 2020; Wang X. et al., 2021).

In this study, we constructed new dual-transgenic BiFC vectors, referred to as *pDTQ* vectors (*pDTQs*), derived from the previously published *pDT1* vector (He et al., 2016b). *pDTQs* allow simultaneous expression of two BiFC proteins within a single vector, with the two proteins controlled under individual promoters and terminators. To facilitate BiFC analysis, we incorporated four different fluorescent proteins into our approach, namely, EYFP, mVenus, mRFP1Q66T (Jach et al., 2006), and mCherry. In total, we crafted eight *pDTQ* vectors, each featuring splitted fluorescent proteins fused in varying orientations. We have successfully validated the applicability of these *pDTQs* in transient expression experiments conducted in both tobacco and rice. This promising outcome underscores the potential utility of *pDTQs* across a diverse spectrum of plant species, including tropical varieties.

## 2 Materials and methods

### 2.1 Construction of BiFC vectors

**FIGURE 1:**
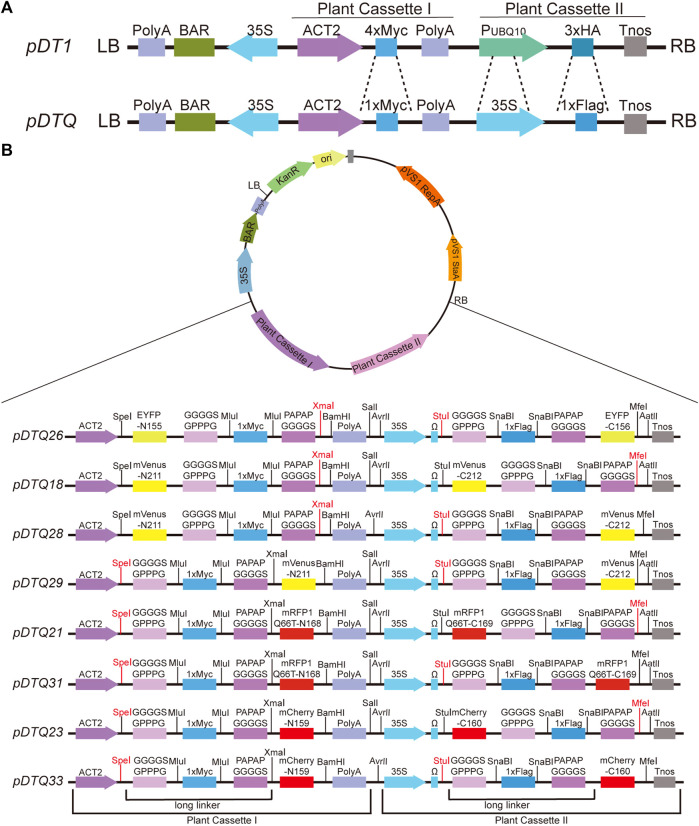
Schematic diagrams of the *pDTQ* vectors. **(A)** Diagrams showing the differences of *pDT1* and *pDTQ* vectors. *pDT1* diagrams was amended form (He Z. et al., 2016). **(B)** Diagrams of the *pDTQ* BiFC vectors. Two long linkers, Plant Cassette I and Plant Cassette II were indicated below the diagrams. The enzyme cutters can be used for gene insertion were highlighted in red.

We employed the *pDT1* vector as the foundational framework (Figure 1A) (He Z. et al., 2016). The 4×Myc tag within the Plant Cassette I was substituted with 1×Myc, accompanied by the incorporation of flexible and rigid linkers on the flanks (GGGGSGPPPG and PAPAPGGGGS) (Robinson and Sauer, 1998; Zhao et al., 2008; Reddy Chichili et al., 2013; Li et al., 2016). In the Plant Cassette II, the *UBQ10* promoter was replaced with the 35S promoter, augmented by the inclusion of an Omega (Ω) sequence to enhance transcription efficiency. Additionally, the 3×HA tag was substituted with 1×Flag, again accompanied by flexible and rigid linkers on the both flanks (GGGGSGPPPG and PAPAPGGGGS). The N- and C-terminal fragments of various fluorescent proteins were inserted into the Plant Cassette I and Plant Cassette II, respectively. The coding sequences (CDSs) for these fluorescent proteins, including EYFP, mVenus, mRFP1Q66T, and mCherry, were synthesized by Tsingke (Beijing, China). The sequences of the fluorescent proteins can be found in Supplementary File S1. The codons for the fluorescent proteins used in the BiFC vectors generated in this study were optimized for plant expression and designed to avoid rare plant codons to ensure efficient protein expression in plants. EYFP was split between amino acids 155 and 156, yielding EYFP-N155 and EYFP-C156. Similarly, mVenus was divided between amino acids 211 and 212, resulting in mVenus-N211 and mVenus-C212. mRFP1Q66T was split between amino acids 168 and 169, leading to mRFP1Q66-N168 and mRFP1Q66-C169. Lastly, mCherry was divided between amino acids 159 and 160, producing mCherry-N159 and mCherry-C160. The BiFC vectors created in this study are named as follows: *pDTQ26* (for EYFP BiFC), *pDTQ18/28/29* (for mVenus BiFC), *pDTQ21/31* (for mRFP1Q66T BiFC), and *pDTQ23/33* (for mCherry BiFC).

All plasmids used in this study were generated using In-Fusion Cloning methods (https://www.takarabio.com/products/cloning/in-fusion-cloning). The sequences subcloned into plasmids were verified by Sanger sequencing. The coding sequences (CDSs) of *BIC2* and *CRY2* were amplified either from *Arabidopsis* cDNA or from previous plasmids using PCR. The purified PCR products were subsequently subcloned into *pDTQ29*, *pDTQ31*, and *pDTQ33* BiFC vectors, digested by *Spe* I/*Stu* I through in-fusion. For *pDTQ26* and *pDTQ28*, *Xma* I/*Stu* I digestion was employed; *pDTQ18* used *Xma* I/*Mfe* I digestion, *pDTQ21* and *pDTQ23* utilized *Spe* I/*Mfe* I digestion. Additionally, the luciferase gene was individually integrated into each corresponding vector to serve as a negative control. All restriction enzymes were purchased from New England Biolabs.

### 2.2 Bimolecular fluorescence complementation (BiFC) assay

BiFC assays in *N. benthamiana* were conducted following previously described methods (Chen et al., 2021). For transient expression, Agrobacterium strains (AGL0) carrying the BiFC plasmids were infiltrated into four-week-old *N. benthamiana* leaves at an OD600 of 0.5. The infiltrated *N. benthamiana* plants were kept in dark conditions overnight and then transferred to white light for 48 h. However, for studying CRY2 photobodies, the injected leaves were incubated in the dark for 2 h before microscopic imaging. Subsequently, samples were collected for microscopic imaging.

### 2.3 Rice protoplast isolation and transfection

The seedlings of one-week-old Kitaake, a rice variety frequently employed in research and laboratory settings, were selected for protoplast isolation and transfection experiments (He F. et al., 2016; Wang Q. et al., 2021). In summary, the chosen leaf sections (∼1 g) were cut into 0.5 mm strips with a sharp razor, and all strips were immediately transferred into a 10 mL enzyme solution (10 mM MES (cat # 4,432-31-9, Sigma-Aldrich, United States) KOH, pH 5.7, 3% (w/v) Cellulase ‘Onozuka’R-10 (cat # 181005-02, Yakult Honsha, Japan), 1.5% (w/v) MacerozymeR-10 (cat # 171208-02, Yakult Honsha, Japan), 10 mM CaCl_2_ (cat # 10035-04-8, Sigma-Aldrich, United States), 0.1% BSA (cat # 9048-46-8, Sigma-Aldrich, United States) (w/v) and 0.6 M Mannitol (cat # 69-65-8, Sigma-Aldrich, United States). After vacuum treatment in the dark for 30 min, enzymatic digestion was carried out by gentle shaking (40 rpm/min) at 28°C in dark conditions for 5 h. The enzymatic hydrolysis was then halted by adding 10 mL of W5 buffer (154 mM NaCl (cat # 7647-14-5, Sigma-Aldrich, United States), 125 mM CaCl_2_, 5 mM KCl (cat # 7447-40-7, Sigma-Aldrich, United States), 2 mM MES KOH, pH 5.7), filtered through a 150–250 mesh stainless steel screen. The filtrate was centrifuged at 100 *g* for 2 min. The pelleted protoplasts were resuspended with W5 buffer and kept at 4°C in the dark for 30 min. Subsequently, the protoplasts were collected by centrifugation at 100 *g* for 2 min. The harvested protoplasts were resuspended in 1 mL MMG solution (0.6 M Mannitol, 15 mM MgCl_2_ (cat # 7791-18-6, Sigma-Aldrich, United States), 4 mM MES KOH, pH 5.7) for subsequent polyethylene glycol 4,000(PEG4000, cat # 25322-68-3, Sigma-Aldrich, United States)-mediated transfection.

PEG-mediated transfection was performed as described with some modifications (Wang Q. et al., 2021). Briefly, after mixing 150 μL of freshly isolated protoplasts with 15 μg plasmid DNA, 165 μL of newly prepared 40% PEG solution was added, and the tubes were inverted several times to mix the contents. Following a 10–20 min incubation in the dark, 1 mL W5 solution was added slowly and mixed well by gently inverting the tubes. The protoplasts were collected by centrifugation at 100 *g* for 2 min and then resuspended in 1 mL W5 solution. This step was repeated three times. Finally, the tubes were incubated in the dark at room temperature for 18–24 h.

### 2.4 Microscopic analyses

Microscopic images were captured using a Leica TCS SP8X DLS confocal laser scanning microscope equipped with a HC PL APO CS2 639/1.40 OIL objective. After 3 days of infiltration, the leaf disk was cut off for imaging. The corresponding wavelength was selected to scan and observe the fluorescence of the respective color. EYFP and mVenus were excited by a 514 nm Ar/ArKr laser with a wavelength of 520–550 nm. mRFP1Q66T and mCherry were excited by a 561 nm white light laser with a wavelength of 610–625 nm. Wavelength scans of three regions of interest from three different infiltrated leaves were used for statistical analyses. All images were captured by Leica.

### 2.5 Western blot analyses

Western blot analysis was performed to determine the expression levels of the two different proteins co-expressed in *N. benthamiana*. For each assay, approximately 0.2 g of 3-day infiltrated *N. benthamiana* leaves were used. The infiltrated leaves were ground in liquid nitrogen, and the protocol referenced previous studies (Qu et al., 2020; Chen et al., 2021) was followed. In brief, total proteins were extracted from the samples using extraction buffer containing 50 mM Tris–HCl (pH 7.5), 150 mM NaCl, 1 mM EDTA, 1 mM dithiothreitol (DTT), 1% Triton X-100 (v/v), 10% glycerol (v/v), and 1× Protease inhibitor cocktail (LOT 63675100, Roche, Germany). Primary antibodies used in this study included Anti-Flag (dilution ratio = 1:1,500, no. F1804; Sigma-Aldrich, United States), and anti-BIC2 antibodies. Secondary antibodies used were anti-Mouse-HRP (dilution ratio = 1:1,500, cat # 31430, Thermo Fisher Scientific, United States) and anti-Rabbit-HRP (dilution ratio = 1:15,000, cat # 31460, Thermo Fisher Scientific, United States). Western blots were detected using the Odyssey CLx Infrared Imaging System (Li-Cor Biosciences, Lincoln, NE, United States).

## 3 Results

### 3.1 Design and construction of the *pDTQ* BiFC vectors

Protein-protein interactions (PPI) play a crucial role in cellular signal transduction. The bimolecular fluorescence complementation (BiFC) assay provides a rapid and intuitive method to investigate the localization and interactions of target proteins within living cells. In the conventional approach, the N-terminal and C-terminal fragments of the fluorescent protein are typically expressed using two separate vectors, which depends on the efficiency of co-transformation into the same cells. To overcome these challenges, we have developed novel dual-transgenic BiFC vectors, known as *pDTQs*, derived from the previously published *pDT1* vector (Figure 1A) (He Z. et al., 2016). The *pDTQ* vectors have two independent Plant Cassettes (Plant Cassette I and Plant Cassette II), which enable the sequential expression of two proteins within a single vector. When we initially used *pDT1* for dual expression, we observed very low protein expression from Plant Cassette II. We suspected that this might be due to the weak promoter. To address this issue, we first modified *pDT1* by replacing the promoter in Plant Cassette II with cauliflower mosaic virus 35S promoter (Figure 1A). We also introduced a long linker, composed of a flexible and rigid linker, between the target and the fluorescent proteins (Figure 1B). These long linkers are designed to facilitate the recombination of the fused fluorescent proteins into their active forms. Additionally, we selected four different excitation wavelengths and monomer-type fluorescent proteins for BiFC analysis, including EYFP, mVenus, mRFP1Q66T, and mCherry (Fan et al., 2008; Feng et al., 2017), which improve the versatility of the BiFC system for studying protein interactions. mRFP1Q66T, an enhanced monomeric red fluorescent protein with improved photostability (Jach et al., 2006). The N- and C-terminal fragments of the same fluorescent proteins were inserted into the Plant Cassette I and Plant Cassette II, respectively, in the same *pDTQ* vector. The resulting BiFC vectors have been designated as *pDTQ26* (for EYFP BiFC), *pDTQ18/28/29* (for mVenus BiFC), *pDTQ21/31* (for mRFP1Q66T BiFC), and *pDTQ23/33* (for mCherry BiFC), with splitted fluorescent proteins fused in varying orientations.

### 3.2 Assessment of the *pDTQ* BiFC vectors in tobacco

Next, we assessed the efficiency of the *pDTQs* system by investigating the oligomerization of *Arabidopsis* CRY2 and CRY2-BIC2 interactions in *N. benthamiana*. *Arabidopsis* cryptochrome 2 (CRY2) has been previously reported to undergo blue light-dependent homodimerization (Wang et al., 2016). BIC2 (Blue light inhibitors of Cryptochromes) has been found to interact with photo-activated CRY2 to inhibit CRY2 photo-oligomerization (Wang et al., 2016; Ma et al., 2020). CRY2-CRY2 or CRY2-BIC2 were cloned into the eight *pDTQ* vectors. The luciferase (LUC) gene was introduced into *pDTQs* as negative controls. Transient expression of the *pDTQ* vectors was carried out following a previously published method (Chen et al., 2021).

**FIGURE 2:**
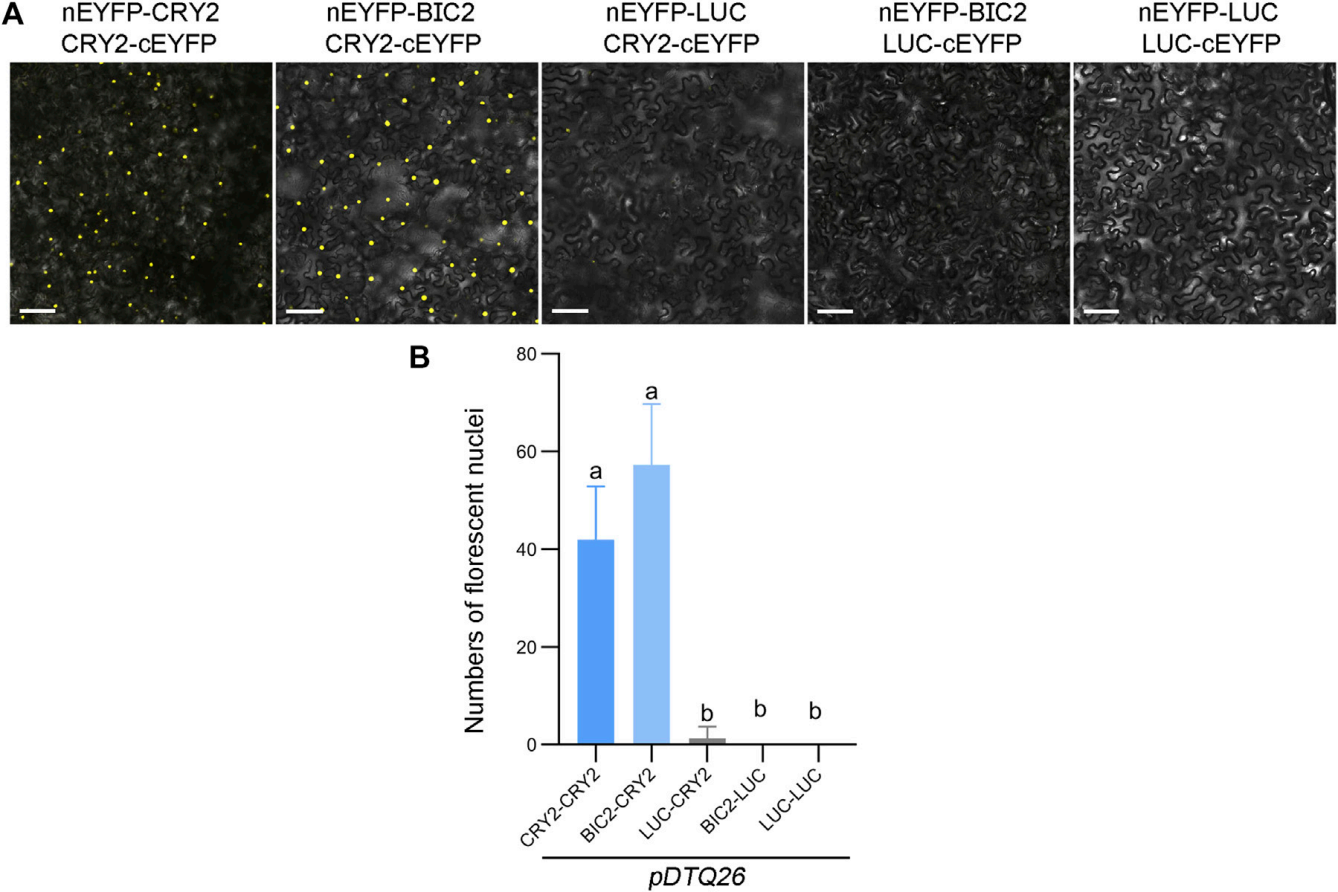
Analysis of CRY2-CRY2 and CRY2-BIC2 interactions by EYFP-based BiFC. **(A)**. The representative confocal images showing the reconstitution of EYFP fluorescence of indicated interacting proteins in *pDTQ26* BiFC vectors. LUC protein served as the negative control. BiFC transient expression experiments were performed in tobacco by injecting Agrobacterium strains (AGL0) carrying the indicated BiFC vector. Fluorescence signals were assessed after 48 h post-transfection by confocal microscope. nEYFP, N-terminus of EYFP (EYFP-N155, as depicted in Figure 1); cEYFP, C-terminus of EYFP (EYFP-C156, as depicted in Figure 1); Scale bar, 100 µm. **(B)**. Quantification of the fluorescent nuclei for indicated BiFC pairs in **(A)** The image from at least three different infiltrated leaves were taken and at least three images were used for quantification. The data are presented as the mean ± SD. Different letters indicate statistically significant differences (*p* < 0.05), as determined by a One-Way ANOVA multiple comparisons test.

To evaluate the efficiency of the *pDTQ26* vector for EYFP-based BiFC analysis, the coding sequence (CDS) of *CRY2* or luciferase (*LUC*) was inserted into the Plant Cassette I via *Xma* I, and the CDS of *CRY2*, *BIC2* or *LUC* was inserted into the Plant Cassette II using *Stu* I. LUC was used as a negative control. BiFC transient expression experiments were conducted in tobacco by injecting Agrobacterium strains carrying the indicated nEYFP and cEYFP plasmids. Fluorescence signals were examined 48 h post-transfection using a confocal microscope. Strong fluorescence signals were detected in the nucleus when nEYFP-CRY2/CRY2-cEYFP and nEYFP-BIC2/CRY2-cEYFP were injected (Figure 2A), indicating the interaction of CRY2-CRY2 and BIC2-CRY2 in plants. Conversely, very few signals were observed in the negative controls (nEYFP-LUC/CRY2-cEYFP, nEYFP-BIC2/LUC-cEYFP, nEYFP-LUC/LUC-cEYFP) (Figure 2A). The number of fluorescent nuclei was quantified for each BiFC pair, with at least three independent injection sites evaluated. The number of fluorescent nuclei in nEYFP-CRY2/CRY2-cEYFP and nEYFP-BIC2/CRY2-cEYFP was significantly higher than that in the negative controls (Figure 2B). Additionally, western blots were performed to assess the protein levels of BIC2, CRY2, and LUC in the transient expression leaves of nEYFP-BIC2/CRY2-cEYFP and nEYFP-BIC2/LUC-cEYFP. Consistent with the observed fluorescence signals, BIC2, CRY2, and LUC proteins were expressed (Supplementary Figure S1A), confirming that the reconstituted fluorescence signals resulted from the expression of BIC2 and CRY2 and their interactions. These results further indicate that the *pDTQ26* vector for EYFP BiFC analysis effectively detects protein-protein interactions.

**FIGURE 3:**
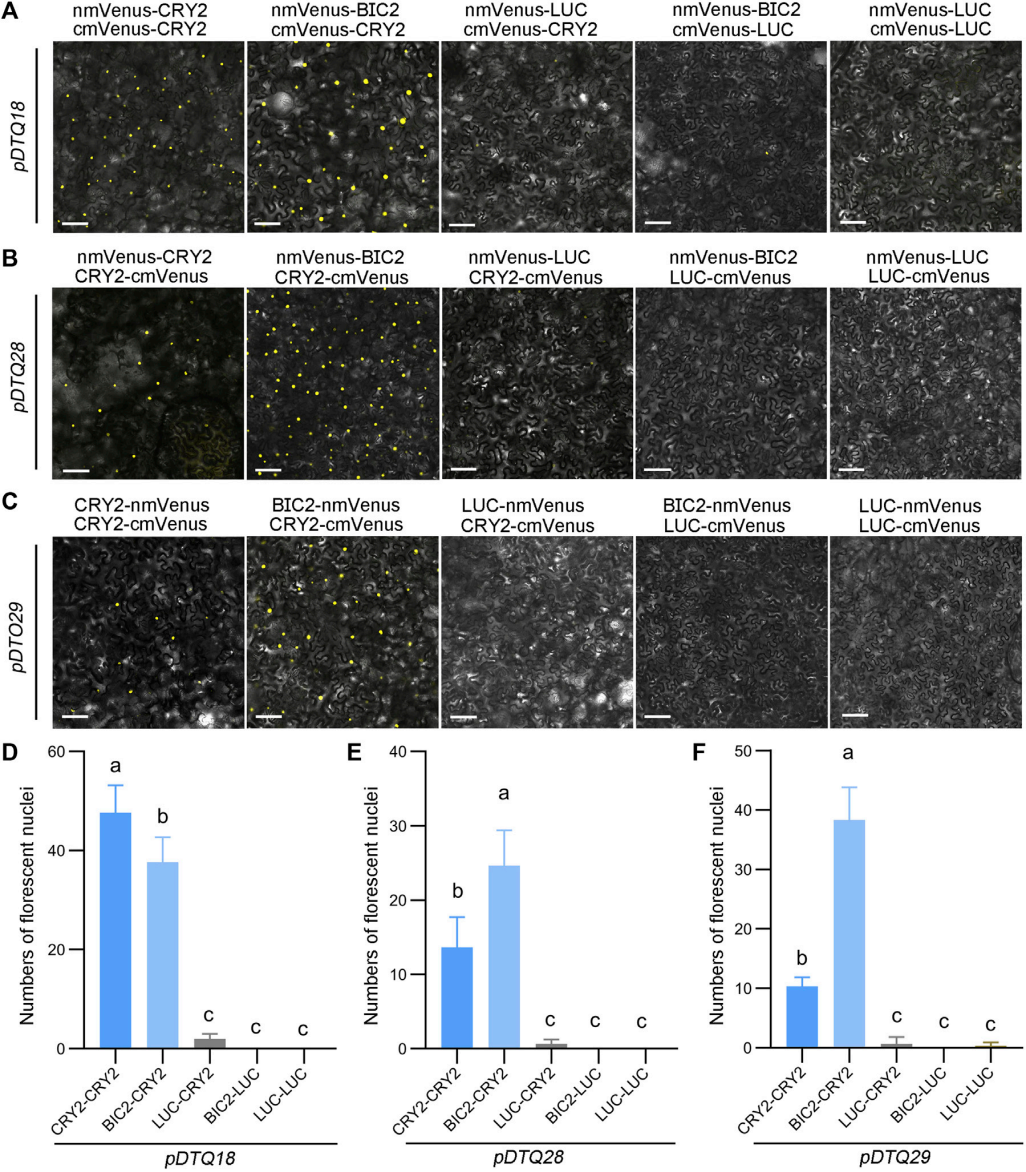
Analysis of CRY2-CRY2 and CRY2-BIC2 interactions by mVenus-based BiFC. **(A–C)**. The representative confocal images showing the reconstitution of mVenus fluorescence of indicated interacting proteins in *pDTQ18* **(A)**, *pDTQ28* **(B)**, and *pDTQ29* **(C)** BiFC vectors. LUC protein served as the negative control. BiFC transient expression experiments were performed in tobacco by injecting Agrobacterium strains (AGL0) carrying the indicated BiFC vector. Fluorescence signals were assessed after 48 h post-transfection by confocal microscope. nmVenus, N-terminus of mVenus (mVenus-N211, as depicted in Figure 1); cmVenus, C-terminus of mVenus (EYFP-C212, as depicted in Figure 1); Scale bar, 100 µm. **(D–F)**. Quantification of the fluorescent nuclei for indicated *pDTQ18/28/29* BiFC pairs in **(A–C)**. The image from at least three different infiltrated leaves were taken and at least three images were used for quantification. The data are presented as the mean ± SD. Different letters indicate statistically significant differences (*p* < 0.05), as determined by a One-Way ANOVA multiple comparisons test.

To assess the effectiveness of the mVenus-based BiFC vectors, we inserted the CDS of *CRY2*, *BIC2*, or *LUC* into the Plant Cassette I of *pDTQ18/28* or *pDTQ29* using *Xma* I or *Spe* I, resulting in constructs with nmVenus-CRY2/BIC2/LUC or CRY2/BIC2/LUC-nmVenus. Additionally, we inserted *CRY2* or *LUC* into the Plant Cassette II of *pDTQ18* or *pDTQ28/29* using *Mfe* I or *Stu* I, creating cmVenus-CRY2/LUC or CRY2/LUC-cmVenus. These plasmids were then individually introduced into tobacco leaves. Strong fluorescence signals were observed in the CRY2-CRY2 and BIC2-CRY2 BiFC pairs (Figures 3A–C). The number of fluorescent nuclei in the CRY2-CRY2 and BIC2-CRY2 pairs was significantly higher than in the negative controls, including LUC-CRY2, BIC2-LUC, and LUC-LUC (Figures 3D–F). For each vector, we selected a positive experimental group (BIC2-CRY2) and a negative control group (BIC2-LUC) for western blot analysis. The immunoblot results confirmed the expression of the indicated proteins in the transient expression assays (Supplementary Figure S1B–D). These findings demonstrate that the *pDTQ18/28/29* vectors for mVenus BiFC analysis are suitable for detecting protein-protein interactions in plants.

**FIGURE 4:**
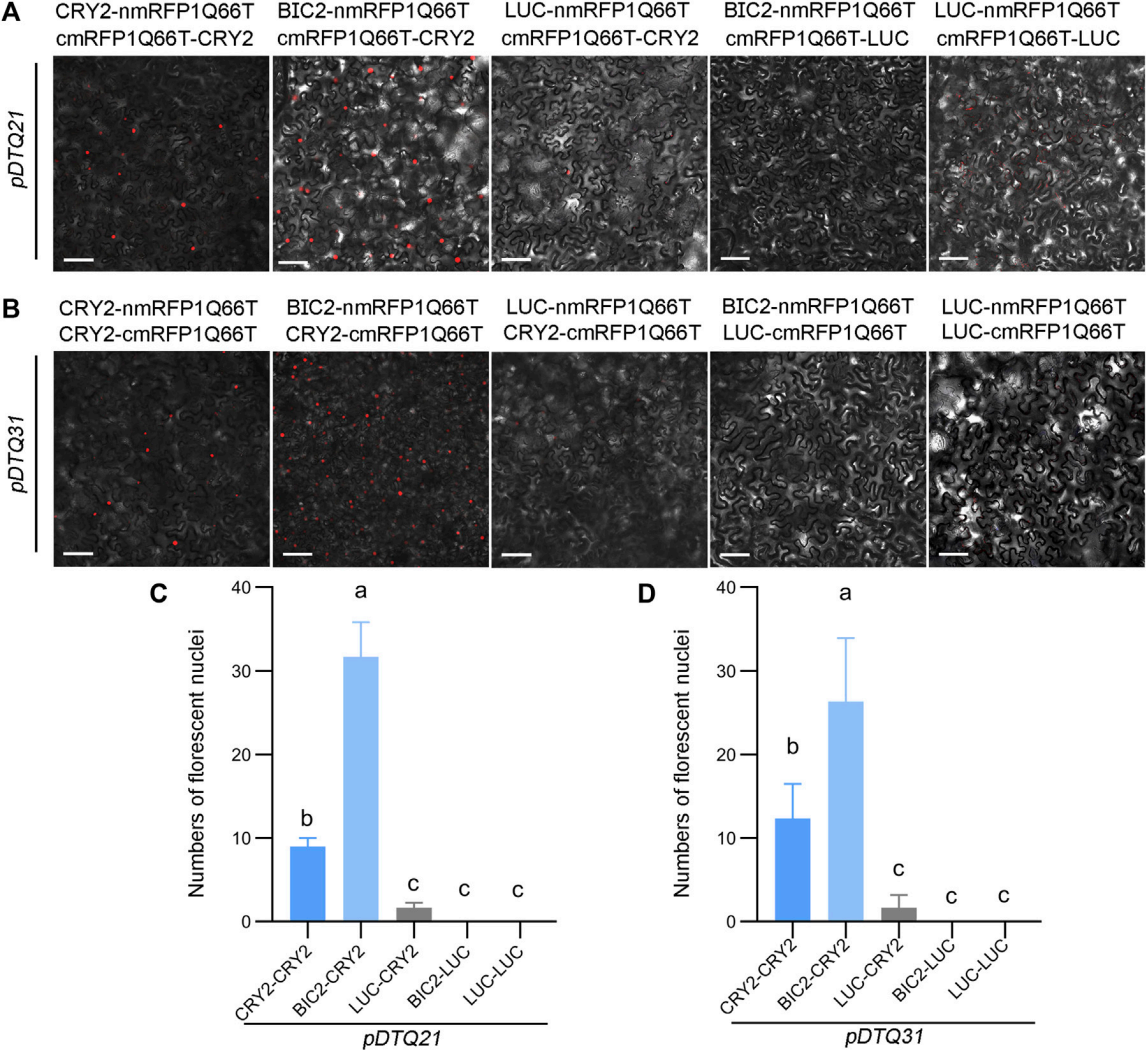
Analysis of CRY2-CRY2 and CRY2-BIC2 interactions by mRFP1Q66T-based BiFC. **(A)** and **(B)**. The representative confocal images showing the reconstitution of mRFP1Q66T fluorescence of indicated interacting proteins in *pDTQ21* **(A)** and *pDTQ31* **(B)** BiFC vectors. LUC protein served as the negative control. BiFC transient expression experiments were performed in tobacco by injecting Agrobacterium strains (AGL0) carrying the indicated BiFC vector. Fluorescence signals were assessed after 48 h post-transfection by confocal microscope. nmRFP1Q66T, N-terminus of mRFP1Q66T (mRFP1Q66T-N168, as depicted in Figure 1); cmRFP1Q66T, C-terminus of mRFP1Q66T (mRFP1Q66T-C169, as depicted in Figure 1); Scale bar, 100 µm. **(C)** and **(D)**. Quantification of the fluorescent nuclei for indicated *pDTQ21/31* BiFC pairs in **(A)** and **(B)**. The image from at least three different infiltrated leaves were taken and at least three images were used for quantification. The data are presented as the mean ± SD. Different letters indicate statistically significant differences (*p* < 0.05), as determined by a One-Way ANOVA multiple comparisons test.

We also explored the use of the red fluorescent protein mRFP1Q66T for BiFC analysis. mRFP1Q66T is known to be monomeric and exhibit increased photostability (Jach et al., 2006). To assess its suitability for BiFC, we split mRFP1Q66T between amino acids 168 and 169, and inserted each N- (amino acids 1–168) and C-terminus (amino acids 169–226) of mRFP1Q66T into the *pDTQ* vectors, resulting in *pDTQ21* and *pDTQ31*. We then inserted the CDS of *CRY2*, *BIC2*, or *LUC* into the Plant Cassette I of *pDTQ21/31* using *Spe* I, creating constructs with CRY2/BIC2/LUC-nmRFP1Q66T. Additionally, we inserted *CRY2* or *LUC* into the Plant Cassette II of *pDTQ21* or *pDTQ31* using *Mfe* I or *Stu* I, resulting in cmRFP1Q66T-CRY2/LUC or CRY2/LUC-cmRFP1Q66T. Fluorescent RFP signals were detected in the CRY2-CRY2 and BIC2-CRY2 BiFC pairs (Figures 4A,B), indicating successful reconstitution of RFP in plants. However, it is worth noting that the CRY2-CRY2 mRFP1Q66T BiFC signals were relatively lower compared to those of EYFP and mVenus BiFC signals, because of fewer average fluorescent nuclei observed (Figures 4C,D). To confirm protein expression, we conducted western blot analysis for the positive experimental group (BIC2-CRY2) and the negative control group (BIC2-LUC), confirming the protein expression of BIC2, LUC, and CRY2 (Supplementary Figure S1E,F). These results demonstrate that the *pDTQ21/31* mRFP1Q66T BiFC vectors can also be effectively used for PPI analysis in plants.

**FIGURE 5:**
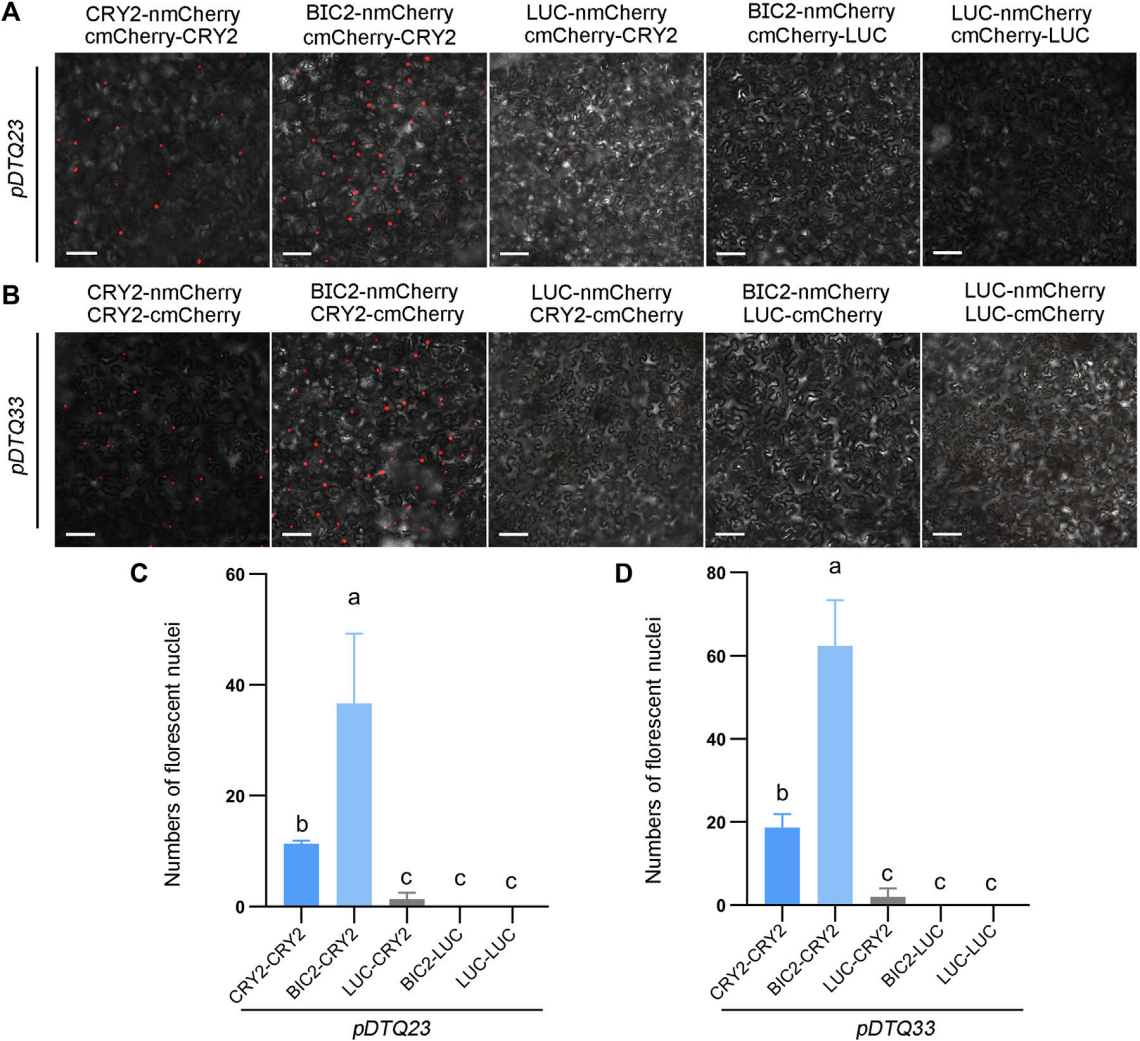
Analysis of CRY2-CRY2 and CRY2-BIC2 interactions by mCherry-based BiFC. **(A)** and **(B)**. The representative confocal images showing the reconstitution of mCherry fluorescence of indicated interacting proteins in *pDTQ23* **(A)** and *pDTQ33* **(B)** BiFC vectors. LUC protein served as the negative control. BiFC transient expression experiments were performed in tobacco by injecting Agrobacterium strains (AGL0) carrying the indicated BiFC vector. Fluorescence signals were assessed after 48 h post-transfection by confocal microscope. nmCherry, N-terminus of mCherry (mCherry-N159, as depicted in Figure 1); cmCherry, C-terminus of mCherry (mCherry-C160, as depicted in Figure 1); Scale bar, 100 µm. **(C)** and **(D)**. Quantification of the fluorescent nuclei for indicated *pDTQ23/33* BiFC pairs in **(A)** and **(B)** The image from at least three different infiltrated leaves were taken and at least three images were used for quantification. The data are presented as the mean ± SD. Different letters indicate statistically significant differences (*p* < 0.05), as determined by a One-Way ANOVA multiple comparisons test.

In addition, we explored another red fluorescent protein mCherry for BiFC analysis. To evaluate the efficacy of the mCherry-based BiFC vectors, we inserted the CDS of *CRY2*, *BIC2*, or *LUC* into the Plant Cassette I of *pDTQ23/33* using *Spe* I, resulting in constructs with CRY2/BIC2/LUC-nmCherry. Similarly, we inserted *CRY2* or *LUC* into the Plant Cassette II of *pDTQ23* or *pDTQ33* using *Mfe* I or *Stu* I, generating cmCherry-CRY2/LUC or CRY2/LUC-cmCherry. Fluorescent RFP signals were observed in the CRY2-CRY2 and BIC2-CRY2 BiFC pairs (Figures 5A,B). Similar to the mRFP1Q66T BiFC vectors, the fluorescence signals observed in the CRY2-CRY2 mCherry BiFC pairs were relatively weaker compared to those of EYFP and mVenus BiFC signals (Figures 5C,D). The expression of BIC2, LUC and CRY2 in the positive experimental group (BIC2-CRY2) and a negative control group (BIC2-LUC) were also confirmed by western blot analysis (Supplementary Figure S1G,H). These results indicate that the *pDTQ23/33* mCherry BiFC vectors can also be effectively used for protein-protein interaction analysis in plants.

**FIGURE 6:**
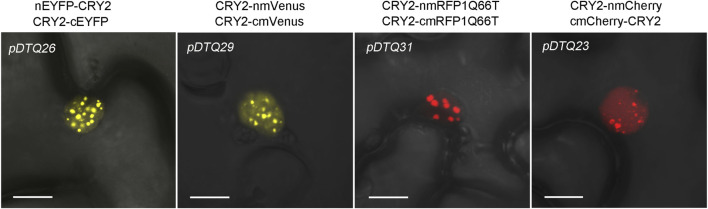
Confocal images showing the CRY2-CRY2 photobodies in indicated BiFC vector transiently expressed in tobacco. Bar, 10 μm.

Given that photoactivated CRY2 has been shown to condense into photobodies in the nucleus (Wang et al., 2016; Wang X. et al., 2021), we conducted a more detailed examination of the photobody formation of CRY2-CRY2 BiFC signals. As depicted in Figure 6, distinct nuclear photobodies were readily observed in the *pDTQ26/29/31/23* constructs for CRY2-CRY2 pairs. Nevertheless, it remains to be determined whether the photobodies formed by CRY2-CRY2 reconstitution from the *pDTQ* vectors exhibit liquid-liquid phase separation, a phenomenon reported for many proteins under specific conditions to enhance local concentration and facilitate their biochemical activities (Yu et al., 2009; Hyman et al., 2014; Shin et al., 2017). An intriguing avenue for further exploration is understanding how proteins are recruited into these foci by their interacting partners. Our *pDTQ* vectors serve as valuable tools for investigating this intriguing question at the visible level. In any case, these results underscore the utility of *pDTQ* vectors in examining the formation of protein foci within plant cells.

### 3.3 Assessment of the *pDTQ* BiFC systems in rice

**FIGURE 7:**
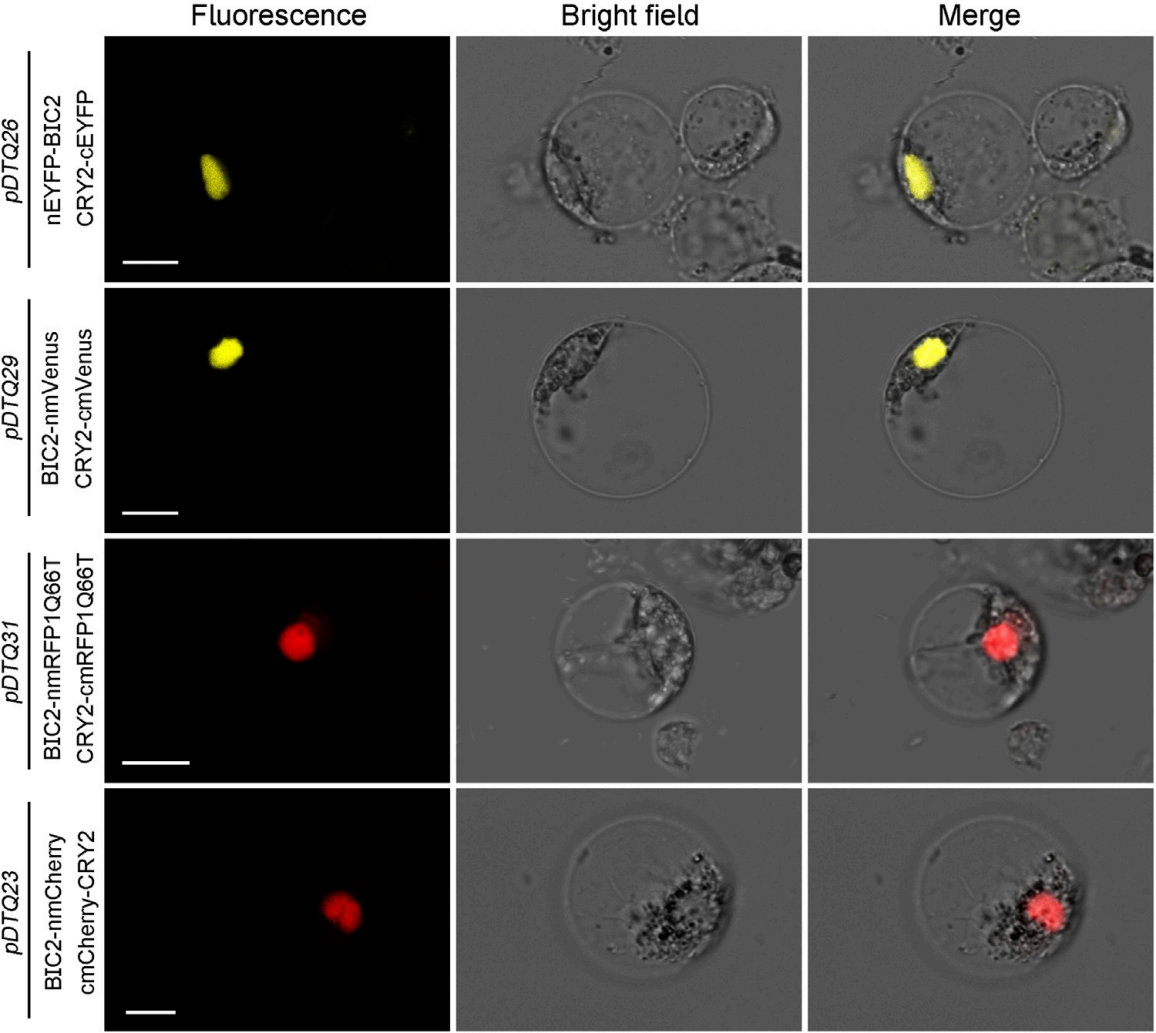
BiFC analysis of AtCRY2-AtBIC2 interaction in rice protoplasts. Indicated BiFC plasmids were transient expressed in rice protoplasts. Fluorescence signals were assessed after 10–12 h post-transfection by confocal microscope. Bar, 10 μm.

The experimentation using the *pDTQ* BiFC vectors has demonstrated high efficiency and low background signal in the tobacco transient expression system. To explore the applicability of *pDTQ* vectors in other plant transient expression systems, particularly in tropical plants, we transiently expressed the BIC2-CRY2 BiFC pair in *pDTQ26*, *pDTQ29*, *pDTQ31*, and *pDTQ23* vectors in rice protoplasts. Strong fluorescence signals were observed in the nuclei of rice protoplasts (Figure 7). Indeed, these results suggest that the *pDTQ* vectors are applicable in the rice transient expression system for testing protein-protein interactions. However, their suitability for other plant species would necessitate further investigation and validation.

## 4 Discussion

The establishment of the dual-transgenic BiFC system stands as a significant advancement in expanding the possibilities of noninvasive fluorescence-based investigations into protein interactions within living organisms. The ability to monitor protein interactions within living cells, while also discerning their subcellular localization, holds immense value for gaining deeper insights into the intricate networks that govern the organization of living cells. Other protein-protein interaction (PPI) methods, such as complementation assays using split-LUC vectors and split-GFP vectors (Magliery et al., 2005; Barnard et al., 2008; Zhao et al., 2008; Chen et al., 2021), are constrained by certain limitations. These include the absence of antibodies for confirming protein expression, the time-consuming and low-efficiency process of co-expression, and the lack of control over the copy number of individual co-expressed target genes. In contrast, our *pDTQ* BiFC vectors offer several distinct advantages over these techniques for assessing protein interactions: 1) Sequential Expression: Our vectors allow for the sequential expression of two proteins within a single vector, significantly increasing transformation efficiency; 2) Long Linker: The inclusion of a long linker between the fluorescent protein fragment and the gene of interest facilitates the recombination of the fused fluorescent protein into an active form; 3) Multiple Fluorescent Proteins: We provide four distinct types of fluorescent proteins—EYFP, mVenus, mRFP1Q66T, and mCherry—for BiFC analysis. This versatility is particularly valuable for studying light-sensitive proteins like photoreceptors; 4) Myc and Flag Tags: The presence of Myc and Flag tags in *pDTQ* vectors enables the direct detection of candidate interacting proteins and allows for co-immunoprecipitation studies; 5) Binary Vectors: All *pDTQ* vectors are binary vectors, facilitating the preparation of stable transgenic plants. This feature simplifies more detailed live interaction studies of interacting proteins within living plants under various conditions. These properties collectively establish *pDTQ* vectors as valuable tools for investigating protein interactions, offering an array of advantages for researchers in the field.

## Data Availability

The datasets presented in this study can be found in online repositories. The names of the repository/repositories and accession number(s) can be found in the article/Supplementary material.

## Supplementary material

SUPPLEMENTARY FIGURE S1 Western blot analysis for the positive experimental group (BIC2-CRY2) and the negative control group (BIC2-LUC). **(A–H)** Immunoblots depict the protein levels of BIC2, CRY2 and LUC in *pDTQ26*(A)/*18*(B)/*28*(C)/*29*(D)/*21*(E)/*31*(F)/*23*(G)/*33*(H) BiFC vectors respectively. The tobacco leaves injecting Agrobacterium strains carrying the indicated BIC2-CRY2 and BIC2-LUC plasmids were harvested for Western blot. The levels of CRY2 and LUC were detected using anti-Flag antibody. The levels of BIC2 were detected using anti-BIC2 antibody. Rubisco stained with Ponceau S was used as the loading control.

## References

[B1] Aebersold R. Mann M. (2003). Mass spectrometry-based proteomics. Nature 422 (6928), 198–207. 10.1038/nature01511 12634793

[B2] Bais P. Alidrissi L. Blilou I. (2023). “Detecting protein–protein interactions using bimolecular fluorescence complementation (BiFC) and luciferase complementation assays (LCA),” in Protein-protein interactions: methods and protocols (New York, NY: Springer US), 121–131. 10.1007/978-1-0716-3327-4_12 37450143

[B3] Barnard E. McFerran N. V. Trudgett A. Nelson J. Timson D. J. (2008). Development and implementation of split-GFP-based bimolecular fluorescence complementation (BiFC) assays in yeast. Biochem. Soc. Trans. 36, 479–482. 10.1042/BST0360479 18481985

[B4] Bellón-Echeverría I. Carralot J. P. Del Rosario A. A. Kueng S. Mauser H. Schmid G. (2018). MultiBacMam Bimolecular Fluorescence Complementation (BiFC) tool-kit identifies new small-molecule inhibitors of the CDK5-p25 protein-protein interaction (PPI). Sci. Rep. 8 (1), 5083. 10.1038/s41598-018-23516-x 29572554 PMC5865166

[B5] Bhat R. A. Lahaye T. Panstruga R. (2006). The visible touch: in planta visualization of protein-protein interactions by fluorophore-based methods. Plant methods 2 (1), 12–14. 10.1186/1746-4811-2-12 16800872 PMC1523328

[B6] Chen H. Zou Y. Shang Y. Lin H. Wang Y. Cai R. (2008). Firefly luciferase complementation imaging assay for protein-protein interactions in plants. Plant Physiol. 146, 368–376. 10.1104/pp.107.111740 18065554 PMC2245818

[B7] Chen Y. Hu X. Liu S. Su T. Huang H. Ren H. (2021). Regulation of Arabidopsis photoreceptor CRY2 by two distinct E3 ubiquitin ligases. Nat. Commun. 12 (1), 2155. 10.1038/s41467-021-22410-x 33846325 PMC8042123

[B8] Citovsky V. Lee L. Y. Vyas S. Glick E. Chen M. H. Vainstein A. (2006). Subcellular localization of interacting proteins by bimolecular fluorescence complementation in planta. J. Mol. Biol. 362, 1120–1131. 10.1016/j.jmb.2006.08.017 16949607

[B9] Comai L. (2003). “Coimmunoprecipitation assay for the detection of kinase-substrate interactions,” in Cancer cell signaling: methods and protocols (Berlin, Germany: Springer), 277–284. 10.1093/mp/ssp040 12616727

[B10] Concepcion J. Witte K. Wartchow C. Choo S. Yao D. Persson H. (2009). Label-free detection of biomolecular interactions using BioLayer interferometry for kinetic characterization. Comb. Chem. high throughput Screen. 12, 791–800. 10.2174/138620709789104915 19758119

[B11] Dai J. P. Zhao X. F. Zeng J. Wan Q. Y. Yang J. C. Li W. Z. (2013). Drug screening for autophagy inhibitors based on the dissociation of Beclin1-Bcl2 complex using BiFC technique and mechanism of eugenol on anti-influenza A virus activity. PloS one 8, e61026. 10.1371/journal.pone.0061026 23613775 PMC3628889

[B12] Ehlert A. Weltmeier F. Wang X. Mayer C. S. Smeekens S. Vicente-Carbajosa J. (2006). Two-hybrid protein–protein interaction analysis in Arabidopsis protoplasts: establishment of a heterodimerization map of group C and group S bZIP transcription factors. Plant J. 46 (5), 890–900. 10.1111/j.1365-313X.2006.02731.x 16709202

[B13] Fan J. Y. Cui Z. Q. Wei H. P. Zhang Z. P. Zhou Y. F. Wang Y. P. (2008). Split mCherry as a new red bimolecular fluorescence complementation system for visualizing protein–protein interactions in living cells. Biochem. biophysical Res. Commun. 367 (1), 47–53. 10.1016/j.bbrc.2007.12.101 18158915

[B14] Feng S. Sekine S. Pessino V. Li H. Leonetti M. D. Huang B. (2017). Improved split fluorescent proteins for endogenous protein labeling. Nat. Commun. 8 (1), 370. 10.1038/s41467-017-00494-8 28851864 PMC5575300

[B15] Frutiger A. Blickenstorfer Y. Bischof S. Forró C. Lauer M. Gatterdam V. (2019). Principles for sensitive and robust biomolecular interaction analysis: the limits of detection and resolution of diffraction-limited focal molography. Phys. Rev. Appl. 11 (1), 014056. 10.1103/physrevapplied.11.014056

[B16] Gehl C. Waadt R. Kudla J. Mendel R. R. Hänsch R. (2009). New GATEWAY vectors for high throughput analyses of protein-protein interactions by bimolecular fluorescence complementation. Mol. Plant 2, 1051–1058. 10.1093/mp/ssp040 19825679

[B17] Grinberg A. V. Hu C. D. Kerppola T. K. (2004). Visualization of Myc/Max/Mad family dimers and the competition for dimerization in living cells. Mol. Cell. Biol. 24 (10), 4294–4308. 10.1128/mcb.24.10.4294-4308.2004 15121849 PMC400484

[B18] Han X. Huang X. Deng X. W. (2020). The photomorphogenic central repressor COP1: conservation and functional diversification during evolution. Plant Commun. 1 (3), 100044. 10.1016/j.xplc.2020.100044 33367240 PMC7748024

[B19] He F. Chen S. Ning Y. Wang G. L. (2016a). Rice (Oryza sativa) protoplast isolation and its application for transient expression analysis. Curr. Protoc. plant Biol. 1 (2), 373–383. 10.1002/cppb.20026 30775867

[B20] He Z. Liu B. Wang X. Bian M. He R. Yan J. (2016b). Construction and validation of a dual-transgene vector system for stable transformation in plants. J. Genet. Genomics 43 (4), 199–207. 10.1016/j.jgg.2016.02.005 27157807

[B21] Hu C. D. Chinenov Y. Kerppola T. K. (2002). Visualization of interactions among bZIP and Rel family proteins in living cells using bimolecular fluorescence complementation. Mol. Cell 9 (4), 789–798. 10.1016/s1097-2765(02)00496-3 11983170

[B22] Hyman A. A. Weber C. A. Jülicher F. (2014). Liquid-liquid phase separation in biology. Annu. Rev. Cell Dev. Biol. 30, 39–58. 10.1146/annurev-cellbio-100913-013325 25288112

[B23] Hynes T. R. Yost E. Mervine S. Berlot C. H. (2008). Multicolor BiFC analysis of competition among G protein beta and gamma subunit interactions. Methods 45, 207–213. 10.1016/j.ymeth.2008.06.008 18586104 PMC2688734

[B24] Jach G. Pesch M. Richter K. Frings S. Uhrig J. F. (2006). An improved mRFP1 adds red to bimolecular fluorescence complementation. Nat. Methods 3, 597–600. 10.1038/nmeth901 16862132

[B25] Kerppola T. K. (2006). Design and implementation of bimolecular fluorescence complementation (BiFC) assays for the visualization of protein interactions in living cells. Nat. Protoc. 1, 1278–1286. 10.1038/nprot.2006.201 17406412 PMC2518326

[B26] Kerppola T. K. (2008). Bimolecular fluorescence complementation (BiFC) analysis as a probe of protein interactions in living cells. Annu. Rev. Biophys. 37, 465–487. 10.1146/annurev.biophys.37.032807.125842 18573091 PMC2829326

[B27] Kim M. H. Roh H. E. Lee M. N. Hur M. W. (2007). New fast BiFC plasmid assay system for *in vivo* protein-protein interactions. Cell. Physiology Biochem. 20, 703–714. 10.1159/000110431 17982253

[B28] Lalonde S. Ehrhardt D. W. Loqué D. Chen J. Rhee S. Y. Frommer W. B. (2008). Molecular and cellular approaches for the detection of protein–protein interactions: latest techniques and current limitations. Plant J. 53 (4), 610–635. 10.1111/j.1365-313X.2007.03332.x 18269572

[B29] Lee C. (2007). Coimmunoprecipitation assay. Methods Mol. Biol. 362, 401–406. 10.1007/978-1-59745-257-1_31 17417028

[B30] Lee Y. R. Park J. H. Hahm S. H. Kang L. W. Chung J. H. Nam K. H. (2010). Development of bimolecular fluorescence complementation using Dronpa for visualization of protein–protein interactions in cells. Mol. imaging Biol. 12, 468–478. 10.1007/s11307-010-0312-2 20373040

[B31] Li G. Huang Z. Zhang C. Dong B. J. Guo R. H. Yue H. W. (2016). Construction of a linker library with widely controllable flexibility for fusion protein design. Appl. Microbiol. Biotechnol. 100, 215–225. 10.1007/s00253-015-6985-3 26394862

[B32] Liu S. Li X. Yang J. Zhang Z. (2014). Low false-positives in an mLumin-based bimolecular fluorescence complementation system with a bicistronic expression vector. Sensors 14 (2), 3284–3292. 10.3390/s140203284 24556667 PMC3958255

[B33] Ma L. Wang X. Guan Z. Wang L. Wang Y. Zheng L. E. (2020). Structural insights into BIC-mediated inactivation of Arabidopsis cryptochrome 2. Nat. Struct. Mol. Biol. 27 (5), 472–479. 10.1038/s41594-020-0410-z 32398826

[B34] Magliery T. J. Wilson C. G. Pan W. Mishler D. Ghosh I. Hamilton A. D. (2005). Detecting protein− protein interactions with a green fluorescent protein fragment reassembly trap: scope and mechanism. J. Am. Chem. Soc. 127 (1), 146–157. 10.1021/ja046699g 15631464

[B35] Miller K. E. Kim Y. Huh W. K. Park H. O. (2015). Bimolecular fluorescence complementation (BiFC) analysis: advances and recent applications for genome-wide interaction studies. J. Mol. Biol. 427 (11), 2039–2055. 10.1016/j.jmb.2015.03.005 25772494 PMC4417415

[B36] Miyawaki A. Llopis J. Heim R. McCaffery J. M. Adams J. A. Ikura M. (1997). Fluorescent indicators for Ca2+ based on green fluorescent proteins and calmodulin. Nature 388 (6645), 882–887. 10.1038/42264 9278050

[B37] Mukherjee S. B. Mukherjee S. Frenkel-Morgenstern M. (2022). Fusion proteins mediate alternation of protein interaction networks in cancers. Adv. protein Chem. Struct. Biol. 131, 165–176. 10.1016/bs.apcsb.2022.05.007 35871889

[B38] Ohad N. Yalovsky S. (2010). “Utilizing bimolecular fluorescence complementation (BiFC) to assay protein–protein interaction in plants,” in Plant developmental biology: methods and protocols (Berlin, Germany: Springer), 347–358. 10.1007/978-1-60761-765-5_23 20734272

[B39] Qu G. P. Li H. Lin X. L. Kong X. Hu Z. L. Jin Y. H. (2020). Reversible SUMOylation of FHY1 regulates phytochrome A signaling in Arabidopsis. Mol. plant 13 (6), 879–893. 10.1016/j.molp.2020.04.002 32298785

[B40] Reddy Chichili V. P. Kumar V. Sivaraman J. (2013). Linkers in the structural biology of protein–protein interactions. Protein Sci. 22 (2), 153–167. 10.1002/pro.2206 23225024 PMC3588912

[B41] Robinson C. R. Sauer R. T. (1998). Optimizing the stability of single-chain proteins by linker length and composition mutagenesis. Proc. Natl. Acad. Sci. 95 (11), 5929–5934. 10.1073/pnas.95.11.5929 9600894 PMC34497

[B42] Samal R. Roy P. S. Sahoo A. Kar M. K. Patra B. C. Marndi B. C. (2018). Morphological and molecular dissection of wild rices from eastern India suggests distinct speciation between O. rufipogon and O. nivara populations. Sci. Rep. 8 (1), 2773. 10.1038/s41598-018-20693-7 29426872 PMC5807453

[B43] Shao S. Zhang H. Zeng Y. Li Y. Sun C. Sun Y. (2021). TagBiFC technique allows long-term single-molecule tracking of protein-protein interactions in living cells. Commun. Biol. 4 (1), 378. 10.1038/s42003-021-01896-7 33742089 PMC7979928

[B44] Shin Y. Berry J. Pannucci N. Haataja M. P. Toettcher J. E. Brangwynne C. P. (2017). Spatiotemporal control of intracellular phase transitions using light-activated optoDroplets. Cell 168 (1), 159–171. 10.1016/j.cell.2016.11.054 28041848 PMC5562165

[B45] Susperreguy S. Prendes L. P. Desbats M. A. Charó N. L. Brown K. MacDougald O. A. (2011). Visualization by BiFC of different C/EBPβ dimers and their interaction with HP1α reveals a differential subnuclear distribution of complexes in living cells. Exp. Cell Res. 317 (6), 706–723. 10.1016/j.yexcr.2010.11.008 21122806 PMC3138133

[B46] Turral H. Burke J. Faures J. (2011). Climate change, water and food security. Rome: FAO.

[B47] Wang Q. Yu G. Chen Z. Han J. Hu Y. Wang K. (2021a). Optimization of protoplast isolation, transformation and its application in sugarcane (*Saccharum spontaneum L*). Crop J. 9 (1), 133–142. 10.1038/s41398-021-01259-0

[B48] Wang Q. Zuo Z. Wang X. Gu L. Yoshizumi T. Yang Z. (2016). Photoactivation and inactivation of Arabidopsis cryptochrome 2. Science 354 (6310), 343–347. 10.1126/science.aaf9030 27846570 PMC6180212

[B49] Wang X. Jiang B. Gu L. Chen Y. Mora M. Zhu M. (2021b). A photoregulatory mechanism of the circadian clock in Arabidopsis. Nat. plants 7 (10), 1397–1408. 10.1038/s41477-021-01002-z 34650267

[B50] Yu X. Sayegh R. Maymon M. Warpeha K. Klejnot J. Yang H. (2009). Formation of nuclear bodies of Arabidopsis CRY2 in response to blue light is associated with its blue light–dependent degradation. Plant Cell 21 (1), 118–130. 10.1105/tpc.108.061663 19141709 PMC2648085

[B51] Zhao H. L. Yao X. Q. Xue C. Wang Y. Xiong X. H. Liu Z. M. (2008). Increasing the homogeneity, stability and activity of human serum albumin and interferon-alpha2b fusion protein by linker engineering. Protein Expr. Purif. 61, 73–77. 10.1016/j.pep.2008.04.013 18541441

