# Supplementary material for: Dual-transgenic BiFC vector systems for protein-protein interaction analysis in plants
Source: Front Genet. 2024 Mar 8;15:1355568. doi: 10.3389/fgene.2024.1355568 (PMC10957565; doi:10.3389/fgene.2024.1355568)
Supplement: Supplementary file 1 [file Presentation1.pdf]

1. At-EYFP

ATGGTGTCTAAGGGAGAGGAGCTTTTCACTGGAGTGGTGCCTATCCTTGTTGAGCTTGATGGAGATGTGAA
CGGACATAAGTTCTCTGTGTCTGGAGAGGGAGAGGGAGATGCTACTTACGGAAAGTTGACTTTGAAGTTCA
TCTGTACTACTGGAAAGTTGCCTGTGCCTTGGCCTACTCTTGTGACTACTTTCGGATACGGActtCAGTGTTTC
GCTAGATACCCTGATCATATGAAGCAGCATGATTTCTTCAAGTCTGCTATGCCTGAAGGATACGTTCAGGAGA
GAACTATCTTCTTCAAGGATGATGGAAACTACAAGACTAGAGCAGAGGTGAAGTTCGAGGGAGATACTCTT
GTGAACAGAATTGAGTTGAAGGGAATTGATTTCAAGGAGGATGGAAACATCTTGGGACATAAGTTGGAGTA
CAACTACAACTCTCATAACGTTTATATCATGGCTGATAAGCAGAAGAACGGAATCAAGGTGAACTTCAAGATC
AGACATAACATCGAGGATGGATCTGTGCAGCTTGCTGATCATTACCAGCAGAACACTCCTATCGGAGATGGA
CCTGTGCTTCTTCCTGATAACCATTACCTTTCTTACCAGTCTGCTCTTTCTAAAGATCCTAACGAGAAGAGAGA
TCATATGGTTCTTTTGGAGTTCGTGACTGCTGCTGGAATCACTCTTGGAATGGATGAGCTTTACAAGtga

2. At-mVenus

ATGGTGTCTAAGGGAGAGGAGCTTTTCACTGGAGTGGTGCCTATCCTTGTTGAGCTTGATGGAGATGTGAA
CGGACATAAGTTCTCTGTGTCTGGAGAGGGAGAGGGAGATGCTACTTACGGAAAGTTGACTTTGAAGCTTA
TCTGTACTACTGGAAAGTTGCCTGTGCCTTGGCCTACTCTTGTGACTACTCTTGGATACGGActtCAGTGTTTC
GCTAGATACCCTGATCATATGAAGCAGCATGATTTCTTCAAGTCTGCTATGCCTGAAGGATACGTTCAGGAGA
GAACTATCTTCTTCAAGGATGATGGAAACTACAAGACTAGAGCAGAGGTGAAGTTCGAGGGAGATACTCTT
GTGAACAGAATTGAGTTGAAGGGAATTGATTTCAAGGAGGATGGAAACATCTTGGGACATAAGTTGGAGTA
CAACTACAACTCTCATAACGTTTATATCACTGCTGATAAGCAGAAGAACGGAATCAAGGCTAACTTCAAGATC
AGACATAACATCGAGGATGGAGGAGTGCAGCTTGCTGATCATTACCAGCAGAACACTCCTATCGGAGATGG
ACCTGTGCTTCTTCCTGATAACCATTACCTTTCTTACCAGTCTAAGCTTTCTAAAGATCCTAACGAGAAGAGA
GATCATATGGTTCTTTTGGAGTTCGTGACTGCTGCTGGAATCACTCTTGGAATGGATGAGCTTTACAAGtga

3. At-mRFP1Q66T

atggcttCttctgaggatgttatcaaggagttcatgagattcaaggttagaatggagggttctgttaacggacatgagttcgagatcgaggga
gagggagagggaagaccttacgagggaactcagactgctaagttgaaggttactaagggtggacctcttcctttcgcttgggatatcctttct
cctcagttcACTtacggttctaaggcttacgttaagcatcctgctgatatccctgattacttgaagttgtctttcccagagggattcaagtggga
gagagttatgaacttcgaggatggaggagttgttactgttactcaggattcttctcttcaggatggagagttcatctacaaggttaagttgaga
ggaactaacttcccttctgatggacctgttatgcagaagaagactatgggatgggaggcttctactgagagaatgtacccagaggacggagc
tttgaagggagagatcaagatgagattgaagttgaaggatggaggacattacgatgcagaggttaagactacttacatggctaagaagcct
gttcagcttcctggtgcttacaagactgatatcaagttggatatcacttctcataacgaggattacactatcgttgaacagtacgagagagcag
agggaagacattctactggagcttga

4. At-mCherry

atggtttCtaagggagaggaggataatatggctattatcaaggagttcatgagattcaaggttcatatggagggttctgttaacggacatgag
ttcgagatcgagggagagggagagggaagaccttacgagggaactcagactgctaagttgaaggttactaagggtggacctcttcctttcgc
ttgggatatcctttctcctcagttcAtgtacggttctaaggcttacgttaagcatcctgctgatatccctgattacttgaagttgtctttcccagag
ggattcaagtgggagagagttatgaacttcgaggatggaggagttgttactgttactcaggattcttctcttcaggatggagagttcatctaca
aggttaagttgagaggaactaacttcccttctgatggacctgttatgcagaagaagactatgggatgggaggcttcttctgagagaatgtacc
cagaggacggagctttgaagggagagatcaagcaaagattgaagttgaaggatggaggacattacgatgcagaggttaagactacttaca
aggctaagaagcctgttcagcttcctggtgcttacaacgttaatatcaagttggatatcacttctcataacgaggattacactatcgttgaaca
gtacgagagagcagagggaagacattctactggaggaatggatgagctttataagtga
