# Supplementary figures and images for: Dual-transgenic BiFC vector systems for protein-protein interaction analysis in plants
Source: Front Genet. 2024 Mar 8;15:1355568. doi: 10.3389/fgene.2024.1355568 (PMC10957565; doi:10.3389/fgene.2024.1355568)

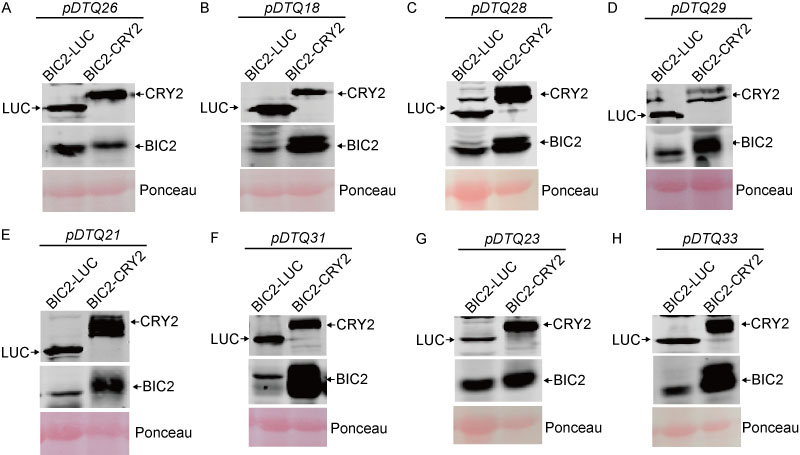

Supplement: Supplementary file 2 [file Image1.jpg]
